# Laparoscopic Resurrection of an Old Technique: A New Approach for Total Urogenital Separation and Rectal Pull-Through in Patients with Long Common Channel Cloacal Malformation

**DOI:** 10.1089/end.2021.0724

**Published:** 2022-08-24

**Authors:** Hebatallah M.A. Taher, Ahmed Fares, Ahmed M.K. Wishahy

**Affiliations:** ^1^ Department of Pediatric Surgery, Cairo University, Cairo, Egypt.; ^2^ Department of Pediatric Surgery, El Fayoum University, El Fayoum, Egypt.

**Keywords:** cloacal malformation, total urogenital separation, laparoscopic pull-through, laparoscopic cloacal repair, laparoscopic vaginal pull-through

## Abstract

**Background and Aims:**

Before the significance of urethral length was highlighted in patients with cloacal malformation, total urogenital mobilization using a posterior sagittal approach was recommended for common channel (CC) length <3 cm, those >3 cm it was followed by urogenital separation. However, many urologists are advocating that the urethral length rather than length of the CC should influence the choice of operation. It is also recommended that total urogenital mobilization should be avoided in patients with short urethral length as intraoperative decision to shift to urogenital separation will risk devascularization of the urethra, advocating total urogenital separation (TUS) from the start; the later technique was deemed difficult using open approach. We describe our experience with laparoscopic TUS and rectal pull-through in patients with cloacal malformation.

**Methods:**

Six patients were operated for a period of 3 years from December 2017 to July 2021; they underwent laparoscopic TUS and rectal pull-through. Preoperative investigations included cystoscopy, genitogram, and MRI pelvis and abdominal ultrasound. IRB approval has been obtained from research ethical committee at Cairo University.

**Results:**

Six female patients born with single perineal opening had colostomy at birth. Age during the second operation ranged from 1 to 4 years. Length of the CC ranged between 2 and 5 cm. Proximal urethral length ranged between 0.5 and 1.5 cm and vaginal depth >3 cm. Average operative time was 4.25 hours. Postoperative period was 1–5 days and uneventful. On the long-term follow-up. No patient developed urethrovaginal fistula and one patient developed vaginal stenosis. All patients had no urinary problems, dry over 4-hour interval, voiding spontaneously, and had normal kidney functions.

**Conclusions:**

Laparoscopic urogenital separation, as well as vaginal and rectal pull-through for cloacal malformation, is feasible in cloacal malformation providing anatomical repair.

## Introduction

The main challenge of cloacal malformation repair and reconstruction is to create three perineal orifices corresponding to the urologic, gynecologic, and gastrointestinal tracts instead of a common single orifice. The reconstruction aim is rectal disconnection from the urogenital sinus and situating the rectum in the muscle complex, enabling bowel control. As well as maintaining urinary continence, consequently renal function, prospective fertility, and future sexual function.

Hardy Hendren^1,2^ has published his study on management of cloacal malformation in early 1980s and he described that total urogenital separation (TUS) was ideal for cloacal malformation with high confluence (long common channel (CC) and vagina opening into urogenital sinus above the urethral sphincter). He advocated that the length of the remaining urethra should determine the definitive repair and proceeding with open TUS, separating the vagina from the urogenital sinus to gain extra length for the urethra and maintaining continence. In contrast, cloacal malformations with short confluences and adequate long remaining urethra he used advancement flaps to create the vaginal opening.

The technique of TUS in cloacal reconstruction has been resurrected in recent publications^3,4^ where authors focus on the length of the proximal urethra when deciding on the nature of operation, either open TUS or total urogenital mobilization (TUM). TUS is ideal for maintaining continence but technically demanding and TUM, which was introduced in 1997, using posterior sagittal approach was originally adapted to overcome the technical difficulty of TUS, especially during the disconnection of the vagina from the common urogenital tract, hence starting by TUM as the initial procedure, however, intraoperatively when the TUM is inadequate, as a result urogenital separation had to be carried out after TUM and the urethra would have been dissected both anteriorly and posteriorly.

This would lead to urethral loss because of compromised blood supply due extensive dissection, consequently those patients will need a urinary diversion such as vesicostomy and a future Mitrofanoff procedure for intermittent catheterization.^3^ Even with TUM primary pull-through of the vagina might occasionally fail raising the need to place a conduit using sigmoid substitutes most of the time, prolonging the time of the operation.

The laparoscopic approach has rendered TUS more feasible, less time consuming, and hence providing more physiologic repair compared with TUM. Laparoscopy-assisted repair of cloacal malformations may provide a paradigm shift in surgical treatment for cloacal malformation.

## Methods

**FIG. 1.**
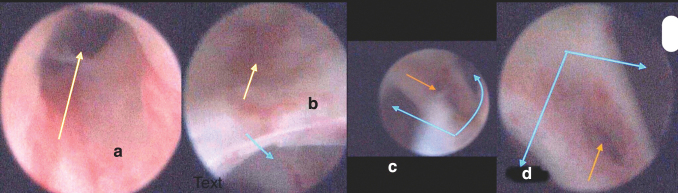
Cystoscopic images of patient 2. **(a, b)** Bladder neck opening (*upward arrow*) and relation of vaginal opening (*downward arrow*) that is relation of confluence to the bladder neck. **(c, d)** View through the vaginal opening showing a vaginal septum between two hemivaginas (*diverging arrows*) in the *middle* of the septum a rectal fistula is seen (*single arrow*). Color images are available online.

A cohort of six patients with cloacal malformation with CC ranging 2–4.5 cm and short urethra ≤1.5 cm underwent laparoscopic complete urogenital separation for a period of 3 years from December 2017 to July 2021. The study obtained the ethical approval of the local ethical committee of our institution. An informed written parental consent has been obtained for all six patients. Investigations included cystoscopy (Fig. 1a–d) and genitogram, which was the mainstay of diagnosis to measure the urethral length and look at the vaginal depth and length, as well as MRI and abdominal ultrasound (US) to delineate associated anatomical abnormalities.

### Operative technique

All surgeries were carried out with the patient positioned supine with legs elevated. For the laparoscopic procedure, pneumoperitoneum was created by an open Hasson method. We used a 5 mm 30° telescope, inserted at the umbilicus. Two working instruments were placed on either side of the umbilicus at the midclavicular line, and a third instrument was used for retraction in the suprapubic area (see Supplementary Movie S1).

**FIG. 2.**
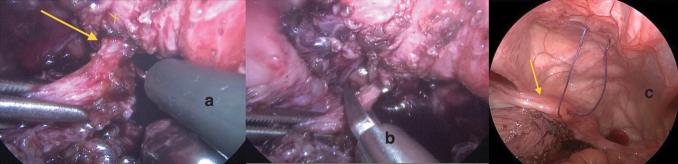
**(a, b)** Intraoperative images patient 5. **(a)** Dissection of rectal fistula (*yellow arrow* off the back of the vagina marked in *yellow cross*), **(b)** application of endoclips to the dissected rectal fistula before cutting. **(c)** Intraoperative image of patient 2 showing a uterus (*yellow arrow*) with a stay stitch applied to the uterus (*yellow arrow*) and another to be applied to bladder to facilitate dissection followed by blunt dissection around connection between vagina and urogenital sinus in the retrovesical space. Color images are available online.

**FIG. 3.**
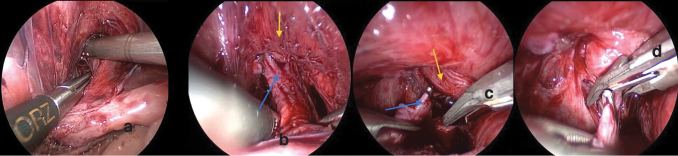
**(a–d)** *Upward arrow* represents vagina and the *downward arrow* represents the bladder. **(c, d)** Endoclip application at the connection between the vagina and bladder before cutting. Color images are available online.

**FIG. 4.**
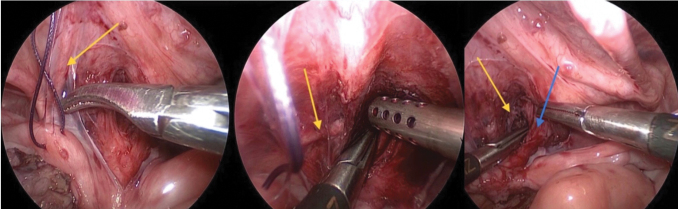
Intraoperative picture of patient 2. Ureteral (*yellow arrow*) course and relation to vagina (*blue arrow*) during vaginal dissection. Color images are available online.

Dissection and separation of the rectal fistula (Fig. 2a–c) followed by dissection of the vagina anteriorly from the urogenital sinus (Fig. 3a–d), by creating a retrovesical space, and then posteriorly in Douglas pouch. Dissection started by hook cautery using low settings on cutting mode, alternating with blunt dissection using Kelly or Maryland forceps (Fig. 3a). Dissection is facilitated by alternate traction on the uterus and bladder by applying stay stitches through the abdominal wall (Fig. 2c). The dissection continued caudally till the confluence with the urethra became visible, and the vagina tapered down at its junction with the urethra (Fig. 3d). Extreme caution was exercised around the vaginal and uterine arteries on both sides as well as ureters (Fig. 4).

**FIG. 5.**
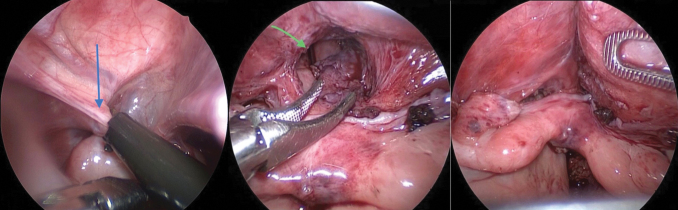
Intraoperative images of patient 2. Round ligament (*blue arrow*) to cut to give extra length to the vaginal intraoperative pictures from patient 2: a grasper introduced from below to grasp the vagina and another instrument, a Maryland forceps, helps guide the vagina through. Pelvic view of bicornuate uterus after being pulled through fashioned introitus. Color images are available online.

**FIG. 6.**
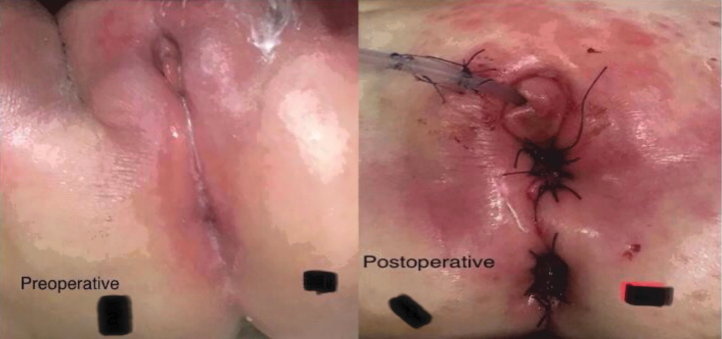
Patient 3. Preoperative and immediate postoperative images. Color images are available online.

Transfixation or clipping of the connection of the vagina to the urogenital channel, followed by cutting (Fig. 3). Clips were initially used to shorten intraoperative time with more experience operators prefer suturing using vicryl 4/0. The uterine round ligament is occasionally cut on either side to allow for easier pull-through of the vagina if extra length is needed (Fig. 5). The tract for the vaginal pull-through is formed through the perineum passing upward until reaching the pelvic cavity. Followed by passage of a clamp through the same tract to grasp the vagina, pull it to the outside and suture it to the skin (Fig. 5). Sufficient vaginal mobilization was achieved in all cases, preserving the vaginal vascularity. There was no need to carry out vaginal wall augmentation using skin flaps (Fig. 6).

## Results

Urethral length was noted and was about 0.5–1.5 cm in length (Table 1). Mean operative time was 4.25 hours with the last operation taking 3 hours duration.

**Table 1.** Preoperative Cystoscopic Measurements of Common Channel and Proximal Urethra, Associated Anomalies as Well as Outcome of the Procedure

Patient no.	Patient 1	Patient 2	Patient 3	Patient 4	Patient 5	Patient 6
Age/years	2 years 6 months	2	1	1	1 year 10 months	4 years
Associated anomaly	Bilateral reflux	None	Mild sacrococcygeal deficiency	Normal	Absent right kidney	Absent left kidney, deformed sacrum
Common channel length/cm	4.5	5	3	3	4	2
Proximal urethral length/cm	0.5	0.5	1.5	1	1	1.5
Operative time/hours	4	4.5	5	4	5	3
Vaginal stricture	yes	No	No	No	No	No
Urethrovaginal fistula	No	No	No	No	No	No
Rectal stricture	No	No	No	No	No	No

Complications (Table 1) included rectal prolapse for patient number 1 for which the patient had a mucosectomy she also had vaginal stenosis. Postoperative there was urine retention in patient 3, which gradually improved on clean intermittent catheterization (CIC) for a period of few weeks. All remaining patients passed urine spontaneously postoperatively and had no retention of urine. All patients were spontaneously voiding and stayed dry >4-hour intervals when assessed over age of two. Follow-up urinary function and US kidneys and bladder were normal. Five patients underwent vaginal dilation with success except patient 1 who has developed stricture with plan to revise when the child reaches puberty. No urinary vaginal fistula was seen in any patient and 5 patients had their stomas reversed and one patient on the waiting list for stoma reversal.

## Discussion

This study reports the early outcomes of laparoscopic TUS and rectal pull-through in six patients with cloacal malformation with CCs ranging 2–4.5 cm and short urethra ≤1.5 followed up for 5 months _3 years after the reconstructive surgery.

Peña stated that in TUM, the initial urogenital sinus mobilization will render the disconnection of the vagina much easily adding that the external urinary sphincter is not essential in urinary continence and urinary incontinence in cloacal patients is because of poor bladder contractility and added that with CCs >3 cm, 70% will need CIC.^5^

In keeping with Hardy Hendren,^2^ we believe that urinary continence requires having a satisfactory bladder volume and normal intravesical pressure, in addition to an adequate bladder outlet resistance to avoid incontinence during normal activity; therefore, it is important to disconnect the vagina from the urogenital sinus and use it to gain extraurethral length improving outlet resistance and maintaining bladder capacity.

Moreover, there are rising questions^3,6,7^ because of the potential of TUM to disrupt the external sphincter. Just the CC length has been the main determinant of TUM, dividing patients into two groups: short CCs <3 cm and long CC >3 cm, some authors suggested^3^ that this classification is overly simplifying the structural problem stating that remaining urethral length after surgery as the most significant factor that should be considered and influence choice of operation.

In a thorough review of literature of the big cloacal repairs series,^3^ there are no records about long-term functional bladder outcome of TUM technique and showing high rates of urinary incontinence using TUM technique. In a study comparing the TUM outcome between two groups. In the first group including 225 patients with a CC length between 1 and 3 cm, 28% of the patients were incontinent to urine, whereas in second group including 175 patients with CC >3 cm, urinary incontinence prevailed in 74% of the patients.^8^ In a different series comprising 50 patients, only 11 patients (about 22% of the series) micturated spontaneously, whereas 12% were kept dry with CIC alone, 46% needed reconstructive urologic surgery and 20% were incontinent.^9^ Rink et al. reported preservation of continence in just one third of their series and the condition of the spine did not change this state, recommending long-term follow-up of this population who underwent TUM procedure as renal function decline may take place in this group.^7^

Based on the literature review and recent cloacal studies a new algorithm based for treatment of cloacal malformation based on urethral length was suggested recommending urogenital separation for urethral length <1.5 cm and TUM for a urethra >1.5 cm in length, provided a CC length short or medium in length that is, <5 cm. Thorough review of literature reveals that the preoperative measurement of the urethral length is essential for deciding the surgical plan while reconstructing the urogenital sinus associated with or without congenital adrenal hyperplasia.^10–12^

The length of the upper urethra depends on the vagina entering above or below external urethral sphincter and is an important factor in the categorization suggested by Rink and colleagues.^13^ This should be the main determinant of the complexity of surgical intervention and outcomes of the patients,^11^ because the upper urethra is a richly innervated structure surrounded completely by circumferential innervations; therefore, authors are advocating against the disruption of the high periurethral area. The mobilization of the pubourethral ligament during TUM could negatively impact continence postoperatively.^11^

Given the high incidence of incontinence associated with TUM, more patients will undergo TUS hence need to master more feasible laparoscopic approach to this technique. The mainstay of urethral measurement in our study was cystoscopy, although other modalities such as MRI and genitography have been used effectively.^10,11,14^

As regard to the technical challenge of separating the vagina from the bladder, the reported laparoscopic technique has the advantage of better observation of the critical anatomical view of the vaginal confluence, as it approaches the neck of the bladder, thus saving the urethral sphincter and surrounding innervations from undue perineal manipulations that can impair the patients' continence. It avoids detachment of the urethral sphincter complex from its fixation to the back of the pubis^9,15^ as in TUM. We believe that mini invasive surgery in narrow spaces such as pelvis is ideal and revolutionized pelvic surgeries and hence there is a role for robotic surgery in repair of cloacal malformations caused by enhanced ergonomics.

It is debated that the high vaginal confluences may need augmentation of the vaginal length with a distal colonic conduit to reach the perineum. However, it was found that dividing the round ligaments (Fig. 5) on either side as described by Fuchs et al. allows an extra length of 2 cm.^12,16^ Moreover, vaginal replacement is still feasible using this approach^15^ and usually reserved for rudimentary or absent vaginas^12^ and in some cases skin flaps could be used.^12^

As described by Pena TUS complications are usually the result of devascularization of the vagina; and comprised urethrovaginal fistula, the most feared complication of this for which a complete reoperation was needed (7%–11%), vaginal stricture (10%), and secondary vaginal atresia (2%).^5^ Vaginal stenosis has been observed in TUS and TUM procedures.^15^ We had no rectovaginal fistulae in our series; however, patient 1 developed vaginal stenosis postoperatively (Table 1), the patient's vaginal depth was >30 mm. Vaginal depth is the distance between the vaginal confluence and perineal point where it should be located is also an important factor as well as the vaginal length and hence important measurements to consider during this procedure, those measurements were studied and applied in patients with congenital adrenal hypeplasia.^11^ Moreover, risk of vaginal devascularization is reduced because of better laparoscopic observation of vaginal blood supply.

Vaginal stenosis, however, is more manageable compared with urinary incontinence and could be addressed at older age during puberty when the estrogenized tissues are more amenable to surgery.^17^ Moreover, the preservation of the perineal body during laparoscopic repair will render this repair easier.^18^

In agreement with Hardy Hendren,^2^ there would be no major psychologic concern in older patients if they need a revision of the vaginal introitus, but if they have no vaginal opening, performing such a major operation at an older age would be a source of major anticipation and anxiety to the teen girl.

Rink and colleagues even recommended early vaginal reconstruction at a young age <6 months, when the child's tissues is under the influence of maternal estrogenic stimulation providing improved vaginal mobilization, hence better recovery and allowing better growth.^19^

Mean operative time was 4.25 hours, the last patient was operated in 3 hours' time has improved throughout the series because of buildup experience. All our patients had no deterioration of renal functions postoperatively nor was there any evidence of upper urinary tract symptoms and were spontaneously voiding and voiding intervals >4 hours period.^7^

What we describe is not a new technique, but the mainstay of cloacal repair before TUM was introduced, which was carried out using open approach, however, was deemed challenging despite its anatomical nature of the repair. Our center's experience with laparoscopic vaginal pull in patients having congenital adrenal hyperplasia associated with a high vaginal confluence and short urethra ≤15 mm, has paved the way for us to laparoscopically revive the original old technique used for surgical management of cloacal malformation, which renders it more feasible because of better observation of the anatomy hence more feasible with better dissection and shorter operative time. In cloacal malformation early genital reconstruction is not as controversial, as in congenital adrenal hyperplasia where there is increasing preference toward delaying such procedures until the child is mature enough to make her own decision.^12^

This study is the biggest single center series of TUS for cloacal repair performed laparoscopically.

## Conclusion

The laparoscopically assisted TUS is a feasible procedure and a viable option in children with cloacal malformation.

## Supplementary Material

Supplemental data

## Abbreviations Used

CC: common channel
CIC: clean intermittent catheterization
MRI: magnetic resonance imaging
TUM: total urogenital mobilization
TUS: total urogenital separation
US: ultrasound
